# Physical activity programs for cardiovascular outcomes in community wheelchair users: A systematic review

**DOI:** 10.3389/fresc.2022.1007778

**Published:** 2022-11-04

**Authors:** Mudasir Saleem Andrabi, Mercy Mumba, Betty Key, Robert Motl

**Affiliations:** ^1^ Capstone College of Nursing, University of Alabama, Tuscaloosa, United States; ^2^ Idda Moffet School of Nursing, Samford University, Birmingham, AL, United States; ^3^ Department of Nutrition and Kinesiology, University of Illinois at Urbana-Champaign, Champaign, IL, United States

**Keywords:** wheelchair users, physical activity, cardiovascular disease, technology use, community

## Abstract

**Purpose:**

Physical inactivity is one of the important factors leading to chronic diseases including cardiovascular disease (CVD) in individuals with disabilities. However, not many Physical Activity (PA) interventions are available for improving the efficacy of PA and cardiovascular outcomes among community wheelchair users. Therefore, this systematic review will appraise the existing PA interventions for the community dwelling wheelchair users; we especially examined features of the PA programs that showed the improvements in PA and the CVD outcomes compared to the interventions that did not show any improvements in these outcomes among these population. The study also aimed to provide some recommendations for future research.

**Materials and Methods:**

A comprehensive and systematic search of literature published between 2015 and 2020 using the databases Scopus, Pubmed, Embase, and Cochrane CENTRAL was conducted. This review has followed the Preferred Reporting Items for Systematic Review (PRISMA) guidelines. The quality of the evidence was assessed by Using Joanna Briggs Institute's critical appraisal tool. Studies that tested the efficacy of PA interventions for community-dwelling adult wheelchair users and published in English were involved. Two reviewers reviewed the literature and any disagreements among these reviewers were resolved by a third reviewer.

**Results:**

Fourteen articles were selected for this review. Most of the studies reported improvements in PA. A few studies followed up the participants and majority of the studies have looked at the CVD outcomes.

**Conclusion:**

Large-scale studies with follow-ups, and community participatory research that evaluates the effect of PA interventions on PA and CVD outcomes among wheelchair users are needed.

## Introduction

In the United States, an estimated 2.7 million adults require the use of a wheelchair based on their physical disabilities (1). Cardiovascular Disease (CVD) is a major cause of morbidity and mortality in these populations (2). There is compelling evidence that wheelchair users with physical disabilities have an increased incidence of chronic diseases, including CVD, cancer, diabetes, and osteoporosis (3). Physical inactivity, unhealthy diet, and alcohol consumption are major risk factors for increased risk of chronic diseases including CVD in wheelchair users (4). The majority of hospitals and clinical facilities focus on short-term rehabilitation services to improve wheelchair user skills for mobility (5). However, after discharge from the medical facilities, the improvement in daily activity in this population remains a concern as there are limited physical rehabilitation services available for continuing the recommended physical activity (PA) in residential communities (6). Additional factors, including disease severity and adaptations, add to the burden of physical inactivity in these individuals. For example, wheelchair users with multiple sclerosis indicated reduced PA participation based on dependence on the mobility device, disease severity (7), and environmental adaptations (8). Half of the wheelchair users with chronic spinal cord injury (SCI) reported no leisure-time PA (6) due to the disease severity**.** This underscores a critical need to focus on PA promotion among wheelchair users to decrease the burden of CVD and improve their overall well-being and quality of life.

The center for disease control (CDC) has recommended moderate to vigorous-intensity PA and muscle strengthening exercises for individuals with disabilities (CDC, 2019). In addition to the reduce risks for chronic disease, PA is essential to regain mobility, improved walking ability, balance, fitness, proper gait, and functional-ability in individuals with disabilities (9). Therefore, these individuals should perform a PA on regular basis that involves bodily movement produced by skeletal muscle contraction resulting in more energy utilization than at resting levels (10).

Wheelchair-users living in community settings engage in insufficient amount of PA for health benefits (3). This may be explained by inaccessible and unaffordable care, low education, and environmental obstacles as critical barriers for engaging in PA (11). This has underscored the importance of designing and delivering feasible and efficacious PA programs for community-dwelling wheelchair users. To that end, we conducted a systematic review to summarize the literature related to PA programs for community-dwelling wheelchair users and understand the features of the PA interventions that improve the PA and CVD outcomes compared to the PA interventions that did not show any improvements in these outcomes for this vulnerable population. We also aimed to identifying gaps in the literature for informing future research.

## Methods

### Overview

#### Literature search strategy

We conducted a comprehensive and systematic search of literature published between 2015 and 2020 using the databases Scopus, Pubmed, Embase, and Cochrane CENTRAL. This time span was selected as most of the research on the efficacy of interventions for PA in wheelchair users was done during this time period. Articles were located using keywords: exercise, physical-activit*, pilot, yoga, sport*, martial-art, recreation, garden, sports, leisure activities, community health services, wheelchairs, wheelchair*, initiative, project, program, plan. These keywords were used as they helped to catch almost all the research studies involving any kind of physical activity interventions for our target population. A professional librarian at the University of Alabama at Birmingham helped with the Literature search. The appendix lists search strings and terms used for different databases (Appendix A).

#### Data extraction and analysis

Two reviewers independently vetted each abstract and full article to ensure the validity and suitability of each study for inclusion. These reviewers have received an intense training in developing systematic reviews and have published some reviews before. Any disagreements among these reviewers were resolved through discussion. If a consensus was not reached, a third party who has expertise in behavioral and physical activity interventions, and has published several reviews, independently reviewed the material and resolved disputes for the articles.

#### Inclusion criteria

Articles describing experimental research were included. This included single subject design, RCTs, multisite RCTs, and pre-post designs of feasibility, pilot, and efficacy studies that examined the effect of PA interventions delivered among community dwelling wheelchair users in the community settings. Database searches were limited to articles written in English and continued until December 20, 2020. Studies that looked at PA for wheelchair users outcome variable, and the population residing in community settings were included for review. Studies involving PA, including leisure time PA, exercises, gardening, sports, recreation, yoga, and martial arts, as well as robotic exoskeletons were included in the review.

#### Exclusion criteria

Studies involving populations below 18 years of age and not involving wheelchair users were excluded. Studies conducted in any setting other than the community settings were excluded. Studies published in any language other than English were excluded.

#### Quality rating

Using Joanna Briggs Institute's critical appraisal tool, the two reviewers assessed the quality of studies selected for this review (12). The tools for critical appraisal were selected based on the study design. A score of 6 was given to the quasi-experimental study and a score of 9 was given to the randomized controlled trials. Any disagreements for critical appraisal were resolved by a third-party consultation.

## Results

**Figure 1:**
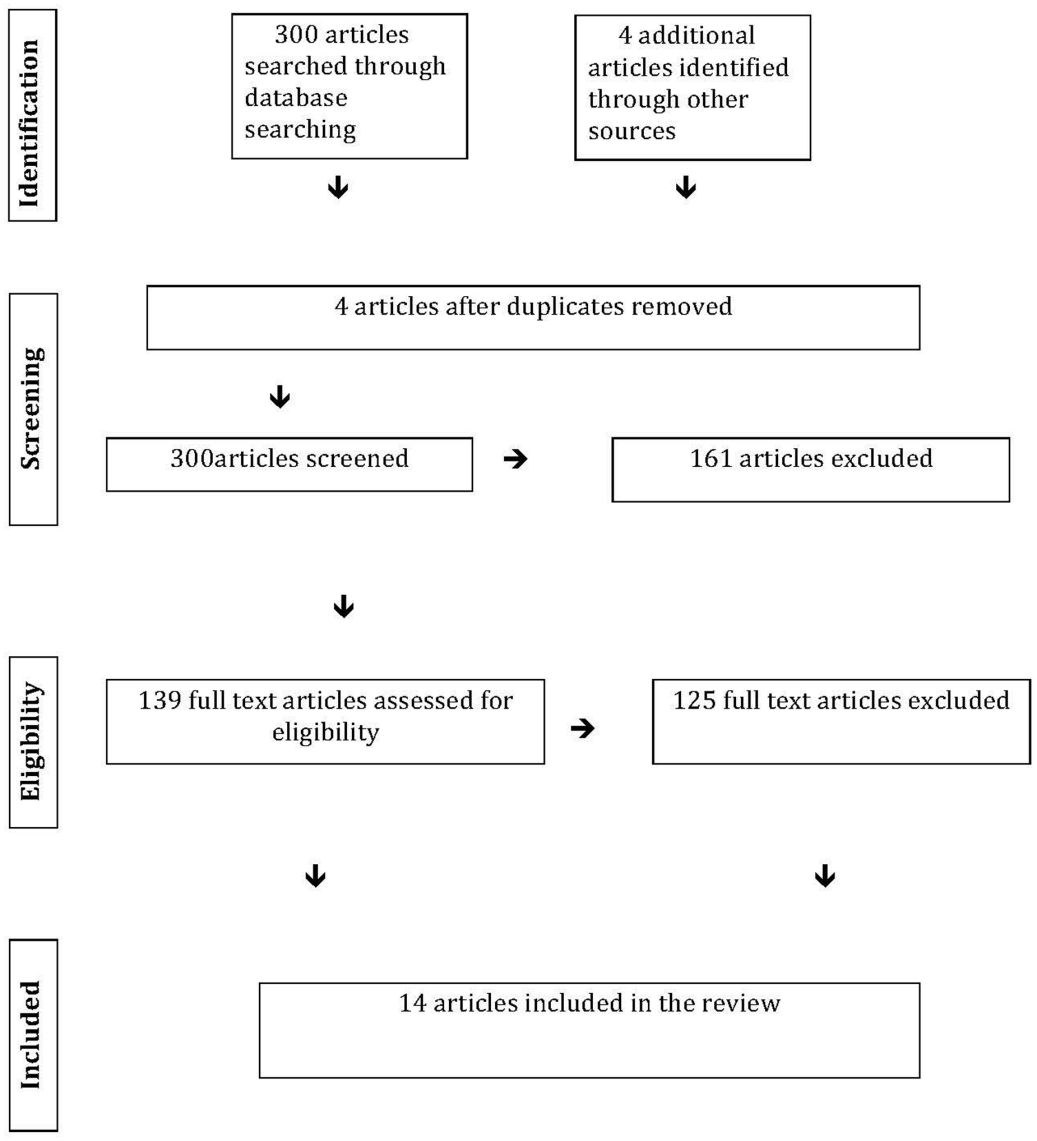
Show the data identification and stepwise data extraction including screening of the articles, removal of duplicates, and selection of the articles based on the inclusion and exclusion criteria.

There were 304 articles identified in our initial search. We removed four duplicate articles. After reviewing the titles and abstracts for the remaining 300 articles, 161 articles were excluded and 139 articles were selected for full text review. After full text review of 139 articles, 125 articles were excluded for the failure to meet criteria, which resulted in the inclusion of 14 articles; Figure 1 provides the flow of article inclusion for this review (PRISMA flow diagram).

Table 1 provides the selected study characteristics. All of the studies were conducted in home/community settings. Study designs include single group intervention design (13, 14), randomized clinical trials (15–22), or multisite randomized clinical trials (23–25).

**Table 1:** Characteristics of the studies (PICO).

Study	Sample/ type of community setting	Study design	Experimental group Intervention type, dose, length	Comparison group intervention	Outcome variables	Results	Follow up
Best et al. (2017)	Manual wheelchair users living in the community (*N* = 38) Setting: Participant homes in community residencies	Randomized controlled trial (Pilot study)	The experimental group received a theory-based intervention that includes the existing PA guidelines for wheelchair users, a toolkit with recommendations for PA, and a mobile phone (with phone number, data plan, and preloaded Facebook page). They received a Smart Phone PA Counseling (SPPAC) program. These sessions were delivered by the trainer *via* mobile phone device. The intervention uses motivational strategies to make the program suitable for the participants’ individual preferences. Dose: The fourteen SPPAC sessions were delivered to participants in 10 weeks and each session was of 30 min duration.	The control group received the PA guidelines same the as that of the experimental group. However, no SPPSC program was delivered to this group.	**PA Outcomes:** - PA measured by actigraphy - Leisure time PA **Others** - PA motivation - Self-efficacy to overcome barriers to PA participation - Anxiety and depression - Satisfaction with the psychological need for PA - Satisfaction for participation in the study - Wheelchair skills - Wheelchair use confidence	Study does not report the results yet.	Ten weeks Three months
Bombardier et al. (2020)	Underactive manual wheelchair user adults with SCI for more than a year with at least 2 cardiometabolic diseases risk factors (*N* = 15) Setting: Participants’ homes or community facilities.	Randomized controlled trial (Feasibility study)	The experimental group receives the treatment *via* phone. It is adapted from a 16-session diabetes prevention program. This is a multi-component intervention that includes a home exercise tool kit (exercise bands with soft grip, ankle strap and door anchor), 16 sessions of PA counseling curriculum by a psychologist *via* phone and a DVD for verbal instructions and a video about stretching, aerobic exercises, and strength training specifically for individuals with paraplegia or tetraplegia. The PA counseling was done by psychologists using motivational interviewing techniques and SMART goal setting to promote the adherence to PA program.	The control group received the usual care control condition. This group received a letter advising them to seek medical care to make lifestyle changes in diet and exercise to address their cardiometabolic risks.	**PA Outcomes:** - PA: minutes/wk for leisure time PA **CV outcomes** - Cardiorespiratory fitness (V_Peak_O) - BMI - Waist circumference - Lean and fat body mass - Insulin sensitivity index - Lipid panel: LDL, HDL **Others:** Depression - Health-related QOL - Pain related to PA and wheelchair use	Post-test at 6 months showed the following non-significant changes: - improvements in MVPA - decrease in depression - increase in pain in both groups but less pain in the experimental group	Six months
Study	Sample/type of community setting	Study design	Experimental group Intervention type, dose, length	Comparison group Intervention	Outcome variables	Results	Follow up
Coulter (2017)	Individuals with SCI (*N* = 24) Setting: participants’ residential homes	Randomized controlled trial (Pilot study)	The experimental group received a web-based physiotherapy for 8 weeks twice a week. Each exercise page has a video, audio, and a written explanation of the exercise. Individual exercise programs (aerobics, strengthening, balance exercises) were prescribed to the experimental group participants according to their abilities. They also completed their online exercise diaries. The physiotherapists delivered the intervention electronically *via* website. They also contacted participants *via* email and phone calls every 2 weeks. Dose: Participants were supposed to perform 30-min exercise sessions twice a week for 8 weeks.	The control group received usual care consisting of self-management of their condition. If these participants were performing any exercises or gym, they were asked to maintain a daily diary and record the exercises and activities that they participated.	**PA Outcomes:** - PA: 6 min push test and 6 min walk test - PA compliance **CV Outcomes:** - Aerobic endurance - HR (work HR and resting HR) **Others:** Acceptance and feasibility of the intervention - Quality of life - Anxiety and depression - Mobility - Perceived exertion - Muscle strength	- A non-significant improvements in the PA of experimental group as indicated by 6 min push test and 6 min walk test - PA compliance was improved in intervention group compared to control group (1.4+_0.8 times/week) - Participants were highly satisfied with the program - Anxiety depression improved in intervention group (effect size = 0.23) - Improvements in pain, strength and quality of life of the experimental group.	None
Divanuglou (2019)	Individuals with SCI (*N* = 7) Setting: Participants residential homes	Prospective cohort study (Protocol for A pilot study)	Active Rehabilitation (AR) training program which is a 7 day program will be delivered to the participants. AR consists of an online wheelchair skills program for wheelchair users	No comparison group	**PA Outcomes:** - Moderate and vigorous PA - Leisure time PA **Others:** - Wheelchair skills test - Wheelchair using confidence and capacity - Self efficacy (personal functioning, social functioning, general self-efficacy) - Life satisfaction - Resilience - Health related QOL	The study does not report the results yet.	Three months
Study	Sample/ type of setting	Study design	Experimental group Intervention type, dose, length	Comparison group intervention	Outcome variables	Results	Follow up
Froehlich-Grobe (2020)	Individuals with immobility. The cause for immobility included SCI, spina bifida, multiple sclerosis, amputation and age related immobility (*N* = 23). Setting: Some intervention sessions were delivered telephonically in participants’ homes and some sessions were in-person in a hospital settings.	Randomized controlled trial with wait list	The experimental group received a Group Lifestyle Balance Program adapted for Individuals with Impaired Mobility (GLB-AIM). It is a 12 month weight loss intervention in which 23 sessions were delivered weekly for 13 weeks, followed by two biweekly sessions. Afterwards monthly sessions were delivered. The session were delivered online and once a month sessions were delivered face to face. The intervention prompts weight loss through reducing calorie intake and by eating healthy diet; It emphasizes walking to increase the PA.	The control group was on waitlist and received the same intervention as the experimental group after 6 months.	**PA Outcomes:** - Walk and wheel time **CV Outcomes:** - Weight - BMI - Waist circumference - BP - Cholesterol - HbA1c **Others:** - Self-efficacy of health behaviors (nutrition, exercise, health promotion, physical well being).	- Experimental group participants had an increase in their MPA minutes of physical activity/ week, whereas it was decreased in waitlist on control group - The experimental group also had increase in walk/wheel time minutes per week. They had a significant decrease in their weight, BMI and waist circumference at 3 months, 6 months and 12 months time point. No changes in these outcomes were seen in the control group at these time points. - Both groups had significant increases in self-efficacy of health behaviors.	Three months Six months Twelve months
Gagnon (2017)	Longterm wheelchair users with SCI (*N* = 14) Setting: Out patient rehabilitation center for community residents	Single group intervention study (Feasibility study)	The participants in experimental group received two familiarization sessions followed by eighteen locomotor training sessions with robotic exoskeleton that were completed in 6–8 weeks. During each training session, participants completed sit-stand transitions, performed quasi-static and dynamic standing balance exercises, and walked with assistance of a rollator walker or with assistance under supervision. These sessions were based on the individual participant's ability to perform these activities. Dose: Participants had two to three 60 min session per week.	No comparison group	**PA Outcomes:** - Standing time, walking time and number of step **Others:** - Perceived motivation to engage in physical activity - Learn to perform sit-to-stand and walk with robotic exoskeleton. - Perceived health benefits, risks and fears associated with engaging in PA. satisfaction with robotic exoskeleton locomotor training	- Participants provided positive feedback for engaging in PA (sit to stand and walking) with exoskeleton. - The standing time, walking time and number of steps were increased by 45.3%, 102.1%, and 248.7% from before to after intervention phase. - health benefits including overall health status, limbic strength and endurance were perceived to be with locomotor training program.	None
Study	Sample/type of community setting	Study design	Intervention type, dose, length	Comparison Group Intervention	Outcome variables	Results	Follow up
Kooijman (2017)	Individuals with SCI for atleast 10 years who are using wheelchair and were physically inactive (*N* = 64) Setting: Participants’ homes in community residential areas	Blinded multicenter Randomized controlled trial	A theory based intervention called “HABITS” was delivered to the experimental group participants. HABITS involves one home visit, five individual and five group sessions delivered in 16 weeks. This intervention facilitates active lifestyle and development of self-skills management by guidance from a counselor, peer support and experiencing task accomplishment to strengthen self-efficacy. These consisting of group meeting, individual counseling and a book. Motivational interviewing was used in counseling the participants.	Control group received information about active lifestyle in a group meeting and a book “How to Stay Fit with SCI.”	. **PA Outcomes:** - Self-reported PA (sports, hobbies, house hold and work related activities) - The amount of self-propelled wheelchair driving measured by using accelerometer **CVD Outcomes:** - Aerobic capacity - BMI **Others:** - Exercise self-efficacy - Proactive coping (dealing with possible future situation) - Social support - Functional independence - Mood - Perceived behavioral control - Exercise self-efficacy - Attitude towards behavior change - Readiness to change with regards to regular exercise - Fatigue - QOL	No significant within and between-group difference was found in any of the primary and secondary outcomes.	- Sixteen weeks - Forty two weeks
Study	Sample/type of community setting	Study design	Intervention type, dose, length	Comparison Group Intervention	Outcome variables	Results	Follow up
Koontz (2020)	Wheelchair users with SCI (*N* = 7). Setting: Participants’ home in community residential areas	Non-randomized clinical trial (pilot study)	Participants received a Hand Cycling High Intensity Interval training (HIIT) with a trainer for 6 weeks, each session comprised of 2–3 min warm ups, followed by ten intervals of cycling with a ratio of one min work at 90% peak power output (PPO) to one min recovery at 0–20% PPO, the two to three min cool down. Dose: HIIT session consisted of two weekly 25-minute sessions.		**PA Outcomes:** - Moderate to vigorous PA, - Adherence to PA sessions - Wheelchair propulsion **CVD Outcomes:** - BMI - HR Aerobic capacity - Oxygen consumption **Others:** - Fatigue - QOL - Satisfaction - Training efficacy - Perceived effectiveness of the training - Endurance - Transferability	- Participants expressed a high level enjoyment - Participants had an increase in their physical capacity HR after receiving the intervention.) - improvements were seen in participants’ training efficacy, endurance, aerobic capacity, transferability, wheelchair propulsion, PA after the intervention. - Extra workout time/ week did not increase - Volume of V_Peak_O increased.	None
Nightangle (2017)	Wheelchair users with SCI for more than a year (*N* = 21) Setting: Participants’ homes in community residential areas	Single blinded multicenter randomized control trial	Home-based moderate intensity upper body exercise intervention is a 6 weeks intervention. The exercise sessions consisted of moderate-intensity arm crank exercise. Dose: The participants received four forty-five-minute exercise sessions per week.	The control group received lifestyle maintenance intervention. They were asked to maintain their habitual PA behavior.	**PA Outcomes:** - Moderate to vigorous PA - Energy intake and expenditure **CV Outcomes:** - VO_2Peak_ - Cardio-respiratory fitness - Sr. fasting Insulin - Body mass - Fat and lean mass - Visceral adipose tissue area - HR - Sr. Triglycerol - Total Cholesterol - HDL - Nonestrified fatty acids - Plasma glucose **Others:** - Health related QOL - Fatigue - Shoulder pain - Exercise self-efficacy	Post-test in six months showed significant improvements in health-related QOL, and fatigue. - Compared to control group, the participants in the experimental group had significant improvements in moderate to vigorous physical activity (with moderate to large effect size) and energy expenditure. - Significant improvement in exercise self-efficacy and PV O2Peak of experimental group. Significant decrease in experimental group participants’ insulin resistance, fasting Sr. insulin concentration, body mass. No changes in any other cardiometabolic disease biomarkers in participants of any of the groups.	None
Study	Sample/ type of community setting	Study design	Intervention type, dose, length	Comparison Group Intervention	Outcome variables	Results	Follow up
(Nooijen (2017)	Wheelchair users with subacute SCI (N39) Setting: The intervention was delivered for 2 months in an in-patient rehabilitation and for rest of the 6 months it was delivered in participants’ homes in community residential areas	Single blinded multicenter randomized controlled trial	The experimental group participants received a regular rehabilitation for 2 months in the inpatient rehabilitation. They also received a behavioral intervention consists of 13 individual face to face sessions with a coach trained in motivational interviewing. The behavioral intervention sessions were delivered twice a month starting before the discharge from inpatient rehabilitation, it continued for 3 months after the discharge. After this one session per month was delivered for three months. Dose: The study does not indicate the duration of the intervention sessions.	Control group only received the regular rehabilitation during their in patient rehabilitation stay. They did not receive any intervention after their discharge from rehabilitation.	**CV Outcomes:** - VO_2Peak_ - BMI - systolic and diastolic blood pressure - lipid profile - total cholesterol, LDL,HDL - triglycerides - glucose levels **Others:** - QOL	- Significant improvements in diastolic blood pressure of the experimental group as compared to the control group. - Between and within the group improvements in BMI, Peak power output, and general health perceptions were seen; however, these changes were not significant. - Significant improvements in cholesterol, LDL in experimental group compared to the control group.	- Six months - Twelve months
Rice (2015)	Wheelchair users with multiple sclerosis (*N* = 14) Setting: Participants’ home, general community setting, and university laboratory	Randomized control trial	The experimental group participants received a regular rehabilitation for 2 months in the inpatient rehabilitation. They also received a behavioral intervention consisting of 13 individual face-to-face sessions with a coach trained in motivational interviewing. The behavioral intervention sessions were delivered twice a month starting before the discharge from inpatient rehabilitation, it continued for 3 months after the discharge. After this one session per month was delivered for three months. Dose: The study does not indicate the duration of the intervention sessions.	The control group were tested for propulsion on treadmill on custom fitted ultralight wheelchair. They did not receive any technique training. They were asked to continue using their primary devices for mobility. Unlike the participants in the intervention group, these participants did not receive any technical training or behavioral intervention. Their PA was measured by the accelerometers like that of the experimental group.	**PA Outcomes:** Activity counts **Others:** - Fatigue - upper extremity strength	- Increased strength significantly in experimental group - decreased fatigue in experimental group but not significant - improved propulsion skills significantly in the experimental group. - An improvement in activity counts in the experimental group than in the control group, however, this difference was not significant.	- Three months
Study	Sample/type of community setting	Study design	Experimental group Intervention type, dose, length	Comparison group intervention	Outcome variables	Results	Follow up
Spungen (2020)	Wheelchair-using veterans with SCI (*N* = 160) Setting: Participants’ homes in community residential areas	Non-blinded randomized control trial	The intervention group received standardized care plus exoskeleton-assisted walking (EAW) advanced training for 30 sessions. The EAW training helped the participants to learn how to be safe and competent to use an exoskeleton for walking at home/community during the intervention phase of 16 weeks. The companions of these participants had to attend at least one-third of these sessions. The participants had to pass the EAW advanced skills test after which they were allowed to take the exoskeleton home and use them for their daily mobility in addition to carrying out the usual Standard of Care (SOC) activities. They also received a multimedia presentation to prevent upper limb pain and fatigue. They were given a social cognitive theory-based behavioral intervention in their homes telephonically by a physical therapist to teach them strategies related to how to maintain the PA. The PA including distance traveled, time spent in propulsion, and propulsion speed was also measured by accelerometer for this group. Dose: The intervention was 8–9 months long including participants pretesting and measurements for device fitting measurements. The study does not specify the time duration for the intervention components including the technical training and telephonic calls.	The control group participants did not receive any EAW advanced training or exoskeleton for their walking. However, they were advised to continue the SOC activities. Like the experimental group, these participants were asked to call for help and guidance if they have any questions or problems. The control group intervention was from 7 to 8 months duration.	–**PA Outcomes:** - Ambulation indoor and outdoor **CV Outcomes:** - Total body fat mass - Sr. lipid profile: low-density lipoproteins, triglycerides, total cholesterol - Insulin resistance **Others:** - QOL - Physical medical health domain: complaints of bladder, bowel, and pain - Mental health summary scores, -Sleep disturbance - Social participation domain - Spinal cord injury functional index	The study has not reported the results outcome measures yet	None
Study	Sample/type of community setting	Study design	Intervention type, dose, length	Comparison group Intervention	Outcome variables	Results	Follow up
Van der Scheer (2016)	Community-dwelling inactive manual wheelchair users with SCI (*N* = 29). Setting: Intervention was delivered in the rehabilitation center	Randomized controlled trial	The experimental/ exercise group received a low-intensity wheelchair training for 16 weeks. This consists of a wheelchair, and treadmill propulsion at 30–40% heart rate reserve or equivalent. The 30 min exercise sessions were delivered twice a week.	The Control group did not receive any intervention.	**PA Outcomes:** - Physical activity measured as metabolic equivalents (MET) using PASIPD (home/week on a 0–180 scale) and propelled distance in a week in the community assessed using an odometer placed on a participants’ daily wheelchair. **Others:** - PO_peak_ - Wheelchair fitness and skills, performance time, ability and strain score.	- No significant effects of the intervention on PO_peak_ of the experimental group. The Control group improved in PO_peak_. - No significant effect of the intervention on wheelchair-specific fitness except P5–15 m. - No significant effects on wheelchair skills performance. - No significant changes in Physical activity levels.	- Two months - Four months
Froehlich-Grobe (2014)	Wheelchair users with sufficient upper arm mobility for arm-based exercises (*N* = 128) Setting: Community residential areas based on the participants’ preferences (participants’ homes, recreation centers)	Randomized controlled trial	Theory-based multi-component exercise intervention. The experimental group or staff-supported group received intensive support from the staff for exercise. These participants received one day of educational information, workshop, and individual exercise plans were developed. Resistance bands, instructions to self-monitor exercise, fifteen regular phone calls and handwritten cards for birthdays, holidays, and major events. They received a monthly newsletter.	The comparison group or the Self-guided group received minimal support from staff; however, these participants received exercise information which was reviewed with them during the first phone call. The rest of the 14 phone calls were made to say thank you and request to return the logs and report about exercise-related injuries. They also received the resistance bands, instructions to self-monitor exercises,, and handwritten cards similar to that of the experimental group	**PA Outcomes:** - Weekly self-reported exercise - PA measured by Accelerometer data **CV Outcomes:** Peak aerobic capacity - Body weight **Others:** - Physical fitness - Predictors for exercise participation	- Experimental group significantly showed greater exercise minutes per week compared to the control group. - Self-reported exercise minutes were moderately correlated with accelerometer data. - No significant between-group differences in aerobic capacity and strength over 12 months. - Both groups had a similar number of exercise logs returned.	- Six months - Twelve months

Abbreviations: PA, Physical Activity; QOL, Quality of life; HR, Heart rate; LDL, Low density Lipoprotein; HDL, High density lipoprotein; MET, Metabolic Equivalent of Task; PASIPD, Physical activity scale for individuals with physical disability; VO_2Peak_, Peak oxygen uptake; BMI, Body Mass Index; SCI, Spinal cord Injury; CV, Cardiovascular.

Ten studies were conducted on wheelchair users with spinal cord injuries (SCI) (13, 14, 16, 17, 20, 21, 23–26), Three studies had participants with immobility due to any disorder (15, 18, 27), one study involved persons with multiple sclerosis (19).

The duration of PA programs ranged between 6 and 12 months. The interventions included exercise interventions (16–18, 23), rehabilitation programs/physiotherapy programs (26), locomotor training programs with robotic exoskeleton (13, 20), hand cycling high intensity interval training (14), low intensity wheelchair training with treadmill propulsion (21), wheelchair and propulsions skills training (19), behavioral interventions including coaching, motivational interviewing (19, 23–25, 28).

These interventions were delivered in different modes including, face-to-face delivery, online delivery, and hybrid of both face-to-face and online sessions. Some of the interventions were delivered among participants individually, some in groups and some both involved both individual and groups sessions. The dose of the PA interventions also varied between these studies. Majority of the studies have delivered interventions weekly once or twice and the duration of a session varied between 25 (1), 30 min (6), 60 min (13). These PA programs are also varied in terms of the total time duration. The shortest intervention was 7 days long (17), and others varied from 6 weeks (24, 29) to a year long intervention (27).

Although the interventions were delivered for the community residential areas; however one of the studies had the intervention delivered in a rehabilitation center near the community residential areas, the participants were coming from their homes to attend the intervention sessions (13). Two studies delivered a component of intervention in the hospital setting and rest of the intervention was delivered in the participants homes (13, 30). All other studies have used participants' homes or community facilities to deliver the interventions in person or online.

The primary outcomes for this review is PA and CVD related outcomes. The secondary outcomes for this review were pain, aerobic endurance, cardiovascular fitness, wheelchair skills, body mass index, motivation, self-efficacy, mood, anxiety and depression, resilience, quality of life, participation and satisfaction with the intervention. Majority of the studies did not have any follow-up after cessation of the programs; three studies had one year follow up (18, 27, 30) and three studies had 3–4 month follow ups (19, 21, 26).

The PA outcomes were assessed differently in these studies, including leisure time PA, moderate to vigorous PA, minutes of walk, wheelchair propelled time and distance, standing time, self-reported PA (sports, hobbies, house hold and work related activities), PA measured as metabolic equivalents. Most of these studies reported improvements in PA (14, 16–19, 24, 27). Some of the studies further reported improvements in exercise self-efficacy (24, 27), depression and anxiety (16, 17), fatigue (19, 24), endurance (14), strength (19) quality of life (24) and the compliance with the PA intervention (17).

Nine studies have looked at the cardiovascular outcomes (1, 16–18, 20, 23, 27, 29, 30). The cardiovascular outcomes that were assessed include, heart rate, blood pressure (systolic and diastolic), body mass index, body weight, serum cholesterol, triglycerides, low-density lipoproteins, high-density lipoproteins, plasma glucose, insulin resistance assessment, cardiorespiratory fitness, Serum fasting insulin, lean and fat mass and visceral adipose tissue area.

Only three studies reported improvements in blood pressure, body mass index (27, 30), heart rate (1) and cholesterol, low density lipoproteins (30).

The studies involved in this review had high quality of methodology, however, due to the small sample size of most of these studies and the inconsistencies in PA measurement, the level of evidence for this review will be of low level.

## Discussion

### Community based PA programs for wheelchair users

Community-based programs for promoting PA for wheelchair users represents a prime opportunity for improving chronic diseases and other outcomes. These programs are scalable and sustainable, yet may not be as effective as supervised center-based programs. Unfortunately, there are not many community-based programs available for wheelchair users living in community settings. The studies included in this review were three multisite randomized controlled trials (RCTs) and seven RCTs, including four pilot RCTs. The majority of RCTs, including one multisite RCT (*n* = 6), reported an increase in PA from before to the after intervention phase; however, some RCTs (*n* = 4) did not report any improvements in PA among wheelchair users. The interventions in these studies differ for instance, behavioral and life style intervention, skills training, low and high intensity exercise training interventions. The interventions further differ in features including type of PA, individual vs. groups sessions, in-person vs. online delivery of intervention, length of whole intervention and duration of intervention sessions, use of technology, theory based vs. no theory-based interventions. These differences in interventions may explain the differences in efficacy of improving the PA among this population.

### Lifestyle and behavioral interventions focusing on self-efficacy and adherence

Lifestyle behavioral interventions involved teaching people skills and strategies for increasing PA as part of daily life and may improve PA among wheelchair users. An increase in PA in a large magnitude (vector counts at wrist) were seen in wheelchair users with multiple sclerosis after receiving a three months behavioral intervention in addition to a wheelchair skills training. This intervention focused on enhancing self-efficacy, overcoming barriers, and identifying facilitators, in addition to the behavioral strategies of self-monitoring, goal setting, planning, optimizing outcome expectations (19). Weekly follow-ups were done telephonically with the participants to teach them about initiation and maintenance of PA. Another behavioral intervention with in-person individual sessions reported a significant improvement in PA and the cardiovascular outcomes of BMI, cholesterol, blood pressure, and low-density lipoproteins at one year follow up. However, these differences were not significant between the experimental and control groups. The intervention had a component of motivational interviewing, that might have increased the adherence to the intervention (25). Hence, the interventions that targeted on improving the PA self-efficacy seem to be promising for these populations. In additions some measures to improve the adherence of intervention must be incorporated into the PA interventions for better efficacy.

Contrary to the above studies, in a multisite RCT, a sixteen-week theory-based intervention that involved a home visit, 5 individual and 5 group counseling sessions and a book did not increase the self-reported PA of wheelchair users in the experimental group. The intervention focused on developing participants' active lifestyle and their self-management skills. There were no within or between-group differences in PA at four- and ten-months follow-ups. The control group had received information about active lifestyle by one group meeting and a book (23). Like other behavioral studies (19, 25), this intervention targeted PA self-efficacy, and behavioral control, however, the baseline self-efficacy in these participants was high, indicating that the intervention was not formulate based on the needs of this population. This could be explaining the ineffectiveness of this intervention on PA. In addition, this study included individuals with SCI for more than 20 years, the authors of this study believe that the long duration of their disabilities of this sample might have made their behavioral tendencies toward inactivity, thereby challenging for them to engage in the PA.

One recent study by Froehlich-Grobe et al. (2020) delivered a 12 month weight loss program to wheelchair users. The intervention was delivered in hybrid form, *via* telephone, and in-person. The intervention had a dietary and a PA component. The waitlist control group received the intervention after six months. Experimental group participants had a significant increase in minutes of walk and wheel time per week, and further had a significant weight loss after six months of starting the intervention. Both groups had a significant increase in self-efficacy of health behaviors (27).

Previously, Froehlich-Grobe et al. (2014) had delivered a theory-based multi-component exercise intervention to experimental and control groups (*N* = 128). The staff support group received additional intensive staff support for exercise, while the self-guided group received minimal support. The staff support group significantly increased exercise (17 min/week) compared to the self-guided group. There was no significant difference in aerobic capacity and strength over twelve month time period (18). Although staff-assisted interventions may show greater efficacy in improving PA, the time and cost related to such interventions affect their sustainability, especially in socio-ecologically deprived communities. Therefore, in order to develop sustainable programs, it is crucial to develop cost-effective interventions, and utilize previously existing resources from communities into these programs.

Community involvement is critical for community participatory research so as to use the already existing resources and develop interventions that are need based and acceptable to a community (31); therefore, it is vital to involve community dwelling wheelchair users when developing health promotion intervention for them. The knowledge about their needs, preferences, resources, barriers and facilities of a community is critical for the development of PA interventions (32). Cole et al. (2019) conducted a feasibility study in which participants' (*N* = 7) opinion related to the content, delivery, and self-management strategies of an evidence-based exercise intervention was assessed. Based on this information, this study developed a six months Workout on Wheels internet intervention (WoWii), which will be evaluated in the future (33). Such community-based research in which community-dwelling wheelchair users are involved in developing PA interventions is scarce. Hence, there is a critical need for such culturally sensitive PA interventions. These interventions may potentially show better acceptability, adherence, and sustainability in community settings as seen in other disciplines (34). Thereby will be more efficacious in improving wheelchair users' PA and their cardiovascular outcome.

The behavioral and lifestyle PA interventions report mixed results with regards to improvements in PA among wheelchair users. Therefore, novel lifestyle behavior programs that are tailored to the needs of these populations should be developed and tested by studies with strong methodology and larger sample sizes. The individual, interpersonal, and environmental levels factors that are found to be related to the adherence to community-based interventions (28) can be incorporated into these programs to improve consumer compliance. Studies should also examine the ecological validity and long-term sustenance of these programs in community settings. In addition, the long-term effects on cardiovascular outcomes are scarcely studied and must be focused in future research.

### Wheelchair skills training programs

Wheelchair skills training programs demonstrated mixed results regarding the changes in PA of community-dwelling wheelchair users. Two RCTs delivered the wheelchair propulsion/skills training interventions to wheelchair users with SCI reported improvements in PA (14, 19). A custom-fit ultra-lightweight manual wheelchair propulsion and skills training revealed an increase in activity counts, strength and propulsion skills, and decreased fatigue of wheelchair users with multiple sclerosis (*n* = 14) compared to the participants in the control group. The experimental group had received theory-based behavioral intervention consisting of the moderate intensity wheelchair skills training and weekly telephonic follow-ups to teach behavioral strategies for initiation and maintenance of PA. Control group participants did not receive any training (19). In contrast, a sixteen-week low-intensity wheelchair propulsion-training program did not show any significant changes in PA levels of the experimental group participants. The participants received twice a week 30-minute sessions. Also, there were no improvements in their wheelchair propulsion and PO_peak_. The study indicates that low-intensity training to be insufficient to improve PA for wheelchair users with long term disabilities (21).

These studies suggest contradicting effects of wheelchair training on PA, and more studies are needed to confirm the efficacy of such interventions on PA of wheelchair users living in community settings. Van der Scheer et al. (2016) had delivered intervention to community dwelling wheelchair users in a rehabilitation setting; the barriers to access for such interventions must be focused especially in relation to availability of these resources in these underserved populations.

### Exercise training programs

In a multicenter RCT, home-based moderate intensity upper body exercise intervention delivered in-person to wheelchair users with chronic SCI (*n* = 21) showed improvements in their PA. This behavioral intervention consisted of a PA component and a dietary component; it involved 45-minute weekly sessions for six weeks. The control group received lifestyle maintenance intervention in which they were asked to maintain their routine PA behavior. The study reported moderate (*d* = 0.62) to large (*d* = 1.37) effect sizes for improvements in PA, cardiorespiratory fitness, and exercise self-efficacy among the intervention participants. These improvements were seen after six weeks of intervention; the study did not do any follow up to see the long-term effects (24). Since the intervention was personalized to each participant's needs and delivered in the home setting, it improved exercise self-efficacy, which is reported to be a key factor in improvements in PA (35, 36). Delivering such interventions that are accessible to the community residents overcomes the barriers of the lack of transportation, time and access. These factors resulted in low attrition (11%) and increased adherence to the intervention, which might explain the large effect size of the intervention outcomes.

Recently, hand cycling high-intensity interval training intervention delivered to the wheelchair users (*n* = 7) showed an increase in the participants' PA. The participants received the intervention in three weekly sessions for six weeks. The participants also showed an increase in their PA heart rate (max.), training efficacy, endurance, aerobic capacity, and wheelchair propulsion skills. The study did not do any follow-ups. The intervention was tailored to each participant's pace and the study used some measure to assess and prompt the intervention adherence (14). These programs can be tested in future studies with large sample size to confirm efficacy in these populations.

### Robotic exoskeleton to improve PA

An eight-week locomotor training program with EKSOTM (version 1.1) robotic exoskeleton was delivered among long-term wheelchair users (*N* = 14) to see its effect on their PA, motivation to engage in PA and performance capability to stand transfers. The intervention consisted of 2–3 training sessions per week in which participants completed sit to stand transitions, quasi static and standing balance exercises, and walked with assistance of a rollator walker while contact-guarded by a physical therapist. The walk with assistance of rollator walker was followed by walk with robotic exoskeleton. There was a significant increase in walking speed after the completion of intervention (*P* < 0.0001) (37). The participants provided positive feedback for the robotic exoskeleton and learning about walking capability and performance of sit-to-stand transfers. The participants' perceived an increase in their motivation to engage in leisure time PA adapted to their condition. In addition, participants also perceived positive effects of this program on their overall health, endurance, and upper limb strength (13).

Another RCT, still in the implementation phase, is planning to deliver exoskeleton assisted walking to experimental group participants (wheelchair users with SCI), in addition to standard care (*N* = 160). The control group is receiving only standard care. The study aims to see the effect of this intervention on indoor and outdoor ambulation, body fat mass, serum lipid profile, insulin resistance, social participation, and sleep disturbance (20). The results of this study are not yet reported. These studies show varied types of locomotor training with varied duration. Therefore, making it difficult to make any conclusions about the dose and duration of the locomotor training interventions. More experimental studies are needed to see the effectiveness of locomotor training with robotic exoskeleton on the PA efficacy of community-dwelling wheelchair users. Studies should be conducted to understand the duration of each type of training and the mechanisms involved in improving PA and cardiovascular health outcomes due to robotic exoskeleton training in these populations.

### Technology based interventions for changing lifestyle PA

Technology offers a way to reach the increased number of populations in less time and helps eliminate the barriers (like lack of transportation and physical therapist time) that interfere with the delivery of care services. The majority of the studies included in this review (*n* = 6) used technology in the interventions. Use of technology (e.g., telephone calls, exercise videos delivered *via* web or DVD, smart phones apps.) in interventions seems to be promising for improving PA efficacy and intervention adherence among wheelchair users (16, 17, 27). Bombardier et al. (2020) adapted and delivered a multipronged diabetes prevention program to the experimental group (*n* = 7). This intervention used the telephone calls for delivering counseling sessions and DVDs videos for delivering aerobic exercise and strength training sessions to the experimental group. The control group participants (*n* = 8) received usual care involving advice to seek preventive care for cardio metabolic risk factors. Posttest at six months indicated though not significant, but there was an increase in experimental group participants' leisure-time PA (minutes of walk per week). There were significant improvements in their exercise self-efficacy and depression levels, also the likelihood of increase pain was significantly low in experimental group (*P* < 0.05) (16). Coulter et al. (2017) reported an improved adherence to web-based physiotherapy intervention in the experimental group participants (*n* = 16). The intervention was delivered for 8 weeks with two sessions per week and was tailored to each participant's individual needs. The intervention consists of strengthening, stretching, aerobic, and balance exercises as appropriate based on participants' abilities. The control group (*n* = 8) were advised to do the self-management for their condition. The participants in experimental group had moderate effect size (*d* = 0.40) improvements in their mobility and endurance. The study also reported improvements in PA compliance and depression in experimental group compared to control group (17). Thereby, increasing their PA performance in everyday life.

These pilot studies suggest that technology-based interventions yield improvements in PA and intervention adherence among wheelchair users, yet studies with large sample size and diverse sample in terms of severity of disability and wheelchair dependence should be conducted to help draw some concrete conclusions. For example, Best et al. (2017) has planned to deliver a 12-week Smart Phone Peer PA Counseling (SPPAC) program *via* phone to the experimental group participants, whereas the control group participants are supposed to receive PA guidelines only. The study aims to improve participants' autonomy to get engaged in leisure time PA as measured by Actigraph, PA self-efficacy and their motivation. The study is reported to be in implementation phase (15). Based on this study, Best et al. (2019) have developed a theory based Active Living Lifestyle Program (ALLWheel) for wheelchair users with SCI. This program is aimed to reach large number of individuals in their communities to improve their self-efficacy, motivation and autonomy, thereby improving their engagement in leisure time PA (38). The intervention is anticipated to have high participant adherence due to the benefits of technology use including flexibility in timing, independence in performing the intervention and lack of need for transportation and scheduling.

These studies have used simple technology (e.g., telephone, web delivery of home exercise videos, DVDs), which can be easily available in rural communities. However, so far, none of the RCTs that have technology-based interventions have been conducted on community dwelling wheelchair users. The use of technology could be useful to overcome the ecological barriers related to remote areas, and their sustenance in these health services deprived areas (39). Therefore, there is a need for studies with controlled designs to test efficacy of technology-based interventions to improve PA and cardio metabolic outcomes in rural dwelling wheelchair users. In addition, theory based and community partnership research should be emphasized to increase the sustenance of these programs.

### Limitations

There is a possibility of some articles not being captured by the search strategies of this systematic review. The above findings should be interpreted carefully as there is a potential bias caused due to the limitations imposed by small sample size of the studies involved in this review. Limitations of this review also arise from the inconsistencies in PA measurement, and other confounding factors.

## Conclusion

The purpose of this review is to gather knowledge about the availability of PA programs focused on improving PA and CVD outcomes among wheelchair users living in community settings. The knowledge gathered from this review illustrates that behavioral and lifestyle interventions have produced promising results in improving PA and CVD outcomes. Although the incorporation of technology that is mostly available to community-dwelling wheelchair users (telephone calls, DVDs, web-based exercise videos) has helped to overcome the facility barriers in community settings, none of the studies were community participatory research. As such, it is critical to involve community resources and partnerships for the ecological validity and sustainability of such PA programs in community settings. Most of the studies are small-scale pilot studies, and only a few have focused on the improvements in CVD outcomes in these populations. Large-scale studies with advanced methodology and long-term follow-ups are required to make any concrete conclusions and interpretations.

## Data Availability

The raw data supporting the conclusions of this article will be made available by the authors, without undue reservation.

## Appendix A

**Embase**

(exercise*:ti,ab OR 'physical activit*':ti,ab OR gymnastic*:ti,ab OR calisthenic*:ti,ab OR run*:ti,ab OR jog*:ti,ab OR swim*:ti,ab OR walk*:ti,ab OR climb*:ti,ab OR 'weight lift*':ti,ab OR pilate*:ti,ab OR qigong:ti,ab OR 'qi gong':ti,ab OR danc*:ti,ab OR 'tai ji':ti,ab OR 'tai chi':ti,ab OR 'tai ji quan':ti,ab OR taiji:ti,ab OR taijiquan:ti,ab OR 'tai chi chuan':ti,ab OR yoga:ti,ab OR sport*:ti,ab OR athletic*:ti,ab OR baseball:ti,ab OR softball:ti,ab OR basketball:ti,ab OR netball:ti,ab OR bicycling:ti,ab OR cycling:ti,ab OR boxing:ti,ab OR cricket:ti,ab OR football:ti,ab OR rugb*:ti,ab OR golf*:ti,ab OR hockey*:ti,ab OR wrestl*:ti,ab OR 'martial art*':ti,ab OR 'hap di do':ti,ab OR judo:ti,ab OR karate:ti,ab OR jujitsu:ti,ab OR 'tae kwon do':ti,ab OR aikido:ti,ab OR wushu:ti,ab OR 'kung fu':ti,ab OR 'gong fu':ti,ab OR gongfu:ti,ab OR mountaineer*:ti,ab OR tennis:ti,ab OR racquetball:ti,ab OR racketball:ti,ab OR 'racket ball':ti,ab OR badminton:ti,ab OR lacrosse:ti,ab OR skating*:ti,ab OR skateboard*:ti,ab OR snowmobiling:ti,ab OR sledding:ti,ab OR skiing:ti,ab OR snowboard*:ti,ab OR soccer:ti,ab OR track:ti,ab OR volleyball:ti,ab OR surfing:ti,ab OR rowing:ti,ab OR polo:ti,ab OR kayaking:ti,ab OR canoeing:ti,ab OR boating:ti,ab OR surfboarding:ti,ab OR recreation*:ti,ab OR ballet:ti,ab OR 'hip hop':ti,ab OR jazz:ti,ab OR tap:ti,ab OR salsa:ti,ab OR fitness:ti,ab OR 'exercise'/exp OR 'sport'/exp OR 'recreation'/exp OR 'fitness'/exp OR 'physical activity'/exp) AND ('wheelchair'/exp OR wheelchair*:ab,ti OR scooter*:ab,ti)

Retrieves 123

**PubMed**

**("Exercise"[Mesh] OR Exercise* [tiab] OR physical-activit*[Title/Abstract] OR pilate*[Title/Abstract] OR yoga[Title/Abstract] OR Sport*[Title/Abstract] OR martial-art [tiab] OR recreation*[Title/Abstract] OR garden*[Title/Abstract] OR "Sports"[Mesh] OR "Recreation"[Mesh] OR "Leisure Activities"[Mesh] OR "Community Health Services"[Mesh]) AND ("Wheelchairs"[Mesh] OR wheelchair* OR scooter*) AND (initiative*[Title] OR project*[Title] OR program*[Title] OR plan*[Title])**

Retrieves 79

**Scopus**

TITLE-ABS-KEY (initiative* OR project* OR program* OR plan*) AND TITLE-ABS-KEY (exercise* OR physical-activit* OR sport* OR recreation* OR leisure) AND TITLE-ABS-KEY (wheelchair* OR scooter*)

Retrieves 94 results

**CENTRAL**

ID Search Hits

#1 MeSH descriptor: [Leisure Activities] explode all trees 18458

#2 MeSH descriptor: [Exercise] explode all trees 24606

#3 MeSH descriptor: [Community Health Services] explode all trees 13872

#4 (Exercise* OR physical-activit* OR pilate* OR yoga OR Sport* OR martial-art OR recreation* OR garden*):ti,ab,kw 128302

#5 (initiative* OR project* OR program* OR plan*):ti 48466

#6 (wheelchair* OR scooter*):ti,ab,kw 848

#7 MeSH descriptor: [Wheelchairs] explode all trees 197

#8 #1 OR #2 OR #3 OR #4145083

#9 #6 OR #7848

#10 #5 AND #8 AND #932

#11 #8 AND #9324
